# Development of acute hydrocephalus does not change brain tissue mechanical properties in adult rats, but in juvenile rats

**DOI:** 10.1371/journal.pone.0182808

**Published:** 2017-08-24

**Authors:** Alice C. Pong, Lauriane Jugé, Lynne E. Bilston, Shaokoon Cheng

**Affiliations:** 1 Neuroscience Research Australia, Margarete Ainsworth Building, Barker Street, Randwick, Sydney, NSW, Australia; 2 University of New South Wales, School of Medical Sciences, Wallace Wurth Building, Kensington, Sydney, NSW Australia; 3 University of New South Wales, Prince of Wales Clinical School, Edmund Blacket Building, Kensington, Sydney, NSW Australia; 4 Macquarie University, Department of Engineering, Faculty of Science and Engineering, Macquarie University, Sydney, NSW, Australia; Biomedical Research Foundation, UNITED STATES

## Abstract

**Introduction:**

Regional changes in brain stiffness were previously demonstrated in an experimental obstructive hydrocephalus juvenile rat model. The open cranial sutures in the juvenile rats have influenced brain compression and mechanical properties during hydrocephalus development and the extent by which closed cranial sutures in adult hydrocephalic rat models affect brain stiffness in-vivo remains unclear. The aims of this study were to determine changes in brain tissue mechanical properties and brain structure size during hydrocephalus development in adult rat with fixed cranial volume and how these changes were related to brain tissue deformation.

**Methods:**

Hydrocephalus was induced in 9 female ten weeks old Sprague-Dawley rats by injecting 60 μL of a kaolin suspension (25%) into the cisterna magna under anaesthesia. 6 sham-injected age-matched female SD rats were used as controls. MR imaging (9.4T, Bruker) was performed 1 day before and then at 3 days post injection. T2-weighted anatomical MR images were collected to quantify ventricle and brain tissue cross-sectional areas. MR elastography (800 Hz) was used to measure the brain stiffness (G*, shear modulus).

**Results:**

Brain tissue in the adult hydrocephalic rats was more compressed than the juvenile hydrocephalic rats because the skulls of the adult hydrocephalic rats were unable to expand like the juvenile rats. In the adult hydrocephalic rats, the cortical gray matter thickness and the caudate-putamen cross-sectional area decreased (Spearman, P < 0.001 for both) but there were no significant changes in cranial cross-sectional area (Spearman, P = 0.35), cortical gray matter stiffness (Spearman, P = 0.24) and caudate-putamen (Spearman, P = 0.11) stiffness. No significant changes in the size of brain structures were observed in the controls.

**Conclusions:**

This study showed that although brain tissue in the adult hydrocephalic rats was severely compressed, their brain tissue stiffness did not change significantly. These results are in contrast with our previous findings in juvenile hydrocephalic rats which had significantly less brain compression (as the brain circumference was able to stretch with the cranium due to the open skull sutures) and had a significant increase in caudate putamen stiffness. These results suggest that change in brain mechanical properties in hydrocephalus is complex and is not solely dependent on brain tissue deformation. Further studies on the interactions between brain tissue stiffness, deformation, tissue oedema and neural damage are necessary before MRE can be used as a tool to track changes in brain biomechanics in hydrocephalus.

## Introduction

Hydrocephalus is characterised by enlarged brain ventricles and compressed brain tissue due to the accumulation of cerebrospinal fluid (CSF) in the ventricles. Although the disease is usually related to CSF obstruction which can be caused by a variety of pathological conditions such as tumour and aqueduct stenosis, hydrocephalus can also develop without any CSF obstruction. In addition, while hydrocephalus usually presents with raised intracranial pressure, it can also occur with normal intracranial pressure. Hydrocephalus therefore has multiple phenotypes, and the pathophysiology, such as the influence of brain mechanical properties during the development of the disease, may differ in these different phenotypes. Brain tissue viscoelastic properties have been shown to change during the development of hydrocephalus[1]. Brain ‘elastance’ inferred from measurements of pressure responses to change in intracranial volumetric content using infusion testing suggest that brain tissue stiffness decreases in Normal Pressure Hydrocephalus (NPH) [2] but increases in acute hydrocephalus [2,3]. Nevertheless, detailed information on the longitudinal changes in brain tissue mechanical properties, the underlying mechanisms for the change in tissue stiffness and how they are associated with change in brain geometry [4–6] remains fragmentary.

Magnetic resonance elastography (MRE) is an imaging technique that measures the mechanical properties of soft biological tissues non-invasively [7,8]. It is a useful technique that can be used to provide spatial maps of tissue stiffness and help to understand how tissue stiffness relates to changes in brain geometry during the development of hydrocephalus. MRE studies have shown that brain tissue mechanical properties in NPH patients are different from healthy humans [9,10] and NPH patients with stiffer brain tissue appear to respond better to shunt treatment [11]. In a recent study, MRE was also used to track changes in brain tissue stiffness immediately after the onset and during the development of acute hydrocephalus in juvenile hydrocephalic rats. Interestingly, the study shows that an increase in brain stiffness was only found in certain brain regions such as the caudate-putamen, and changes in brain stiffness were influenced by multiple factors such as brain tissue deformation and alteration in tissue water content [1]. As the skulls of the juvenile rats were not fused, the cranium expanded during ventricular enlargement and it remains unclear to what extent the open cranial sutures in the juvenile rats influenced the pattern of brain tissue deformation and change in brain tissue stiffness during the disease.

The objective of this study was to determine whether changes in brain stiffness in adult rats (with closed cranial sutures) are different from those in juvenile rats during the development of acute hydrocephalus. Specifically, the aims of this study were to determine, 1) how brain tissue mechanical properties change during hydrocephalus development in adult rats with a fixed cranial volume and 2) how such changes are related to brain tissue deformation. Results from this study were compared with the results from the juvenile hydrocephalic rats study [1].

## Materials and methods

### Experimental procedures

The study protocol was approved by the Animal Care and Ethics Committee of the University of New South Wales (UNSW, Sydney, Australia). Fifteen 10 week old female Sprague-Dawley (SD) rats were purchased from the Animal Resources Centre (Canning Vale, WA, Australia) and acclimatized for a week prior to any interventions. Animals were housed in threes in standard cage, on a 12 hr light/dark cycle. All animals had free access to water and pellet food and were checked and weighed daily. To induce hydrocephalus, rats were anaesthetized with 1.5% isoflurane in 100% oxygen delivered at a constant rate of 1L/min via a face mask. Under aseptic conditions, the tip of a 28 gauge needle was inserted into the cisterna magna and 60 μL of a suspension of kaolin (Sigma-Aldrich, Castle Hill, Australia) 25% w/v in 0.9% saline at the rate of 6 μL/s was injected (hydrocephalic group, n = 9). This volume was twice the amount used for the juvenile rats in our previous study. Based on our preliminary studies, this volume was necessary to create ventriculomegaly in the adult rats similar to the juvenile rats. In addition, following the same protocol, a control group (n = 6) was created by only inserting the 28 gauge needle through the cisterna magna membrane without injecting any suspension. After the surgery, subcutaneous injections of analgesic (Temgesic®, buprenorphine hydrochloride, 0.05 mg/kg, Cenvet, Blacktown, Australia) and 4% glucose solution were given 2–3 times daily or when the animals expressed signs of pain or distress.

### Image acquisition

Imaging was performed using a 9.4T MRI (Bruker BioSpec Avance III 94/20 Ettlingen, Germany) in the Biological Resources Imaging Laboratory of University of New South Wales, Sydney, Australia. Signals were generated and received using a 86 mm ID quadrature volume transmitter in combination with a 20 mm diameter single loop surface coil (Bruker) placed on top of the rat skull.

All animals were imaged at two time points: Baseline (one day pre injection, day -1), and three days post kaolin / sham injection (day 3). These time points were the same as those used for juvenile rats in our previous study [1]. The rats were placed in prone position and anaesthetized with 1–1.5% isoflurane (Pharmachem, Eagle Farm, Australia) delivered at a constant rate of 1L/min. The body temperature and breathing rate of the rats were monitored in real time.

For both time points, the following imaging protocol was performed:

i Anatomical images were acquired using a rapid acquisition with relaxation enhancement (TurboRARE) sequence to localize the foramina of Monro. The parameters were: repetition time / echo time = 9469 ms/9.5 ms, rapid acquisition with relaxation enhancement factor = 16, twelve signal averages, one repetition, 32 contiguous axial slices, field of view = 22.4 mm × 22.4 mm, slice thickness = 1 mm, matrix = 128 × 128, acquisition time = 15 minutes 9 seconds.

The following acquisitions were centered on the foramina of Monro.

ii High resolution anatomical T2-weighted axial images of the ventricular system, cortical gray matter and caudate-putamen were obtained using a two dimensional TurboRARE sequence, with the following parameters: repetition time / echo time = 2000 ms/9.5 ms, rapid acquisition with relaxation enhancement factor 8, twenty four signal averages, one repetition, nine contiguous axial slices, field of view = 22.4 mm × 22.4 mm, slice thickness = 350 μm, matrix = 192 × 192, acquisition time = 19 minutes 12 seconds.
iii MRE data was collected with a mechanical vibration of 800 Hz transmitted to the brain via a teeth bar connected to the incisors [1]. Provided that the waves propagate in full 3D and are not dominated by waves in any given direction (which is the case for this study as waves radiate from the skull inwards throughout the brain), the MRE sequence that we used in our laboratory [12] encodes three-dimensional wave propagation so that data produced using this sequence would not be affected by the location of the transducer and axis of vibration. The following parameters were used for the MRE scan: repetition time/ echo time = 1880 ms /28.75 ms, one signal average, nine contiguous axial slices, field of view = 22.4 mm × 22.4 mm, slice thickness = 350 μm and matrix = 192 × 192. The acquisition time for all three directions was 48 minutes 7 seconds. Images were collected in matched geometry with the high resolution anatomical T2-weighted images.

All animals were euthanized immediately after the MR scans at day three, under deep anaesthesia (2.5%-3% of isoflurane, Pharmachem, Eagle Farm, Australia) by intracardiac perfusion of 100 mL of phosphate buffered saline 1X (Sigma-Aldrich, Castle Hill, Australia) and then 100 mL of 10% buffered formalin solution (Thermo Fisher Scientific, Australia Pty Ltd, Scoresby Vic, Australia).

### Data reconstruction and analysis

**Fig 1:**
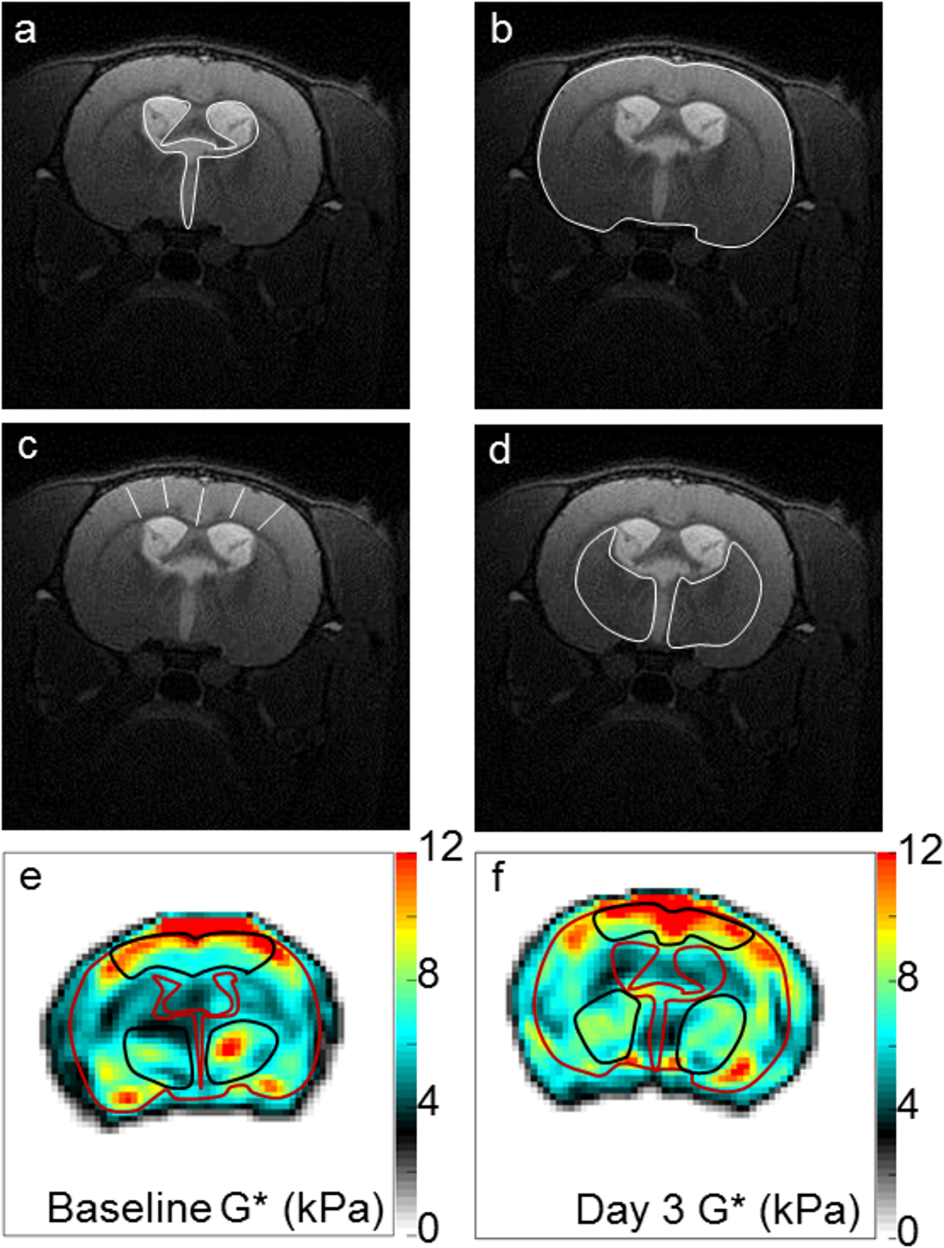
Regions of interest to characterise the effects of hydrocephalus on changes in brain structure size and stiffness in adult rats. Changes in brain structure size were characterised using high resolution anatomical T2-weighted images by quantifying: a) ventricle size (i.e cross-sectional area of the ventricular system), b) head size (i.e cranial cross-sectional area), c) cortical gray matter thickness (from the pial surface to the subcortical white matter averaged over 5 locations) and d) caudate putamen cross-sectional area (sum of the right and left caudate-putamen). Brightness of the images was increased by 20% for visualization. White lines indicate each region of interest in a—d. Brain stiffness was then quantified by calculating the shear modulus (G*, kPa) at e) baseline and in f) day 3 in the cortical gray matter and the caudate putamen (black ROIs) using MRE performed at 800 Hz. For visualization, the outer boundary of the brain tissue and brain ventricle boundaries have been outlined in red on G* maps.

For all rats, high resolution anatomical T2-weighted images were used to quantify a) cross-sectional area of the ventricular system (Fig 1A), b) cranial cross-sectional area (area encompassed in the outer boundary of the brain tissue, Fig 1B), c) cortical gray matter thickness (from the pial surface to the subcortical white matter averaged over five locations, Fig 1C) and d) caudate putamen cross-sectional area (sum of the right and left caudate-putamen, Fig 1D). Analysis was performed on data collected at both time points (baseline and day three post-injection) using ImageJ (version 1.47, National Institutes of Health, USA).

Shear modulus (G*) of the cortical gray matter and caudate putamen in both the hydrocephalic and control rats were also measured at both time points and averaged across the middle three slices. ROIs were drawn manually on the magnitude images of the MRE data and then transferred on the G* map. Brain regions near the ventricles that showed partial volume effects due to voxels containing cerebrospinal fluid were excluded [13]. Representative ROIs on the shear modulus map of the cortical gray matter and caudate-putamen are shown in Fig 1E and 1F respectively. G* was calculated using an in-house MRE inversion software which had been described in detail previously (Green et al., 2008). Signal-to-noise ratio of data analysed was considered appropriate and reliable if the wave amplitude was between 3.8 and 5.8 μm in the cortical gray matter, and between 2.0 and 4.9 μm in the caudate-putamen.

Finally, changes in brain structure size and stiffness during hydrocephalus development in the adult and juvenile rats obtained using the same imaging protocols (4 week old female Sprague-Dawley rats, n = 8 [1]) were compared. In order to enable a meaningful comparison between the adult and juvenile hydrocephalic rats, brain tissue area (cranial cross-sectional area minus the ventricle system cross-sectional area) was calculated at baseline and 3 days post-injection. Brain tissue area measured at day 3 was then normalised to baseline values to obtain the normalised brain tissue area in order to provide a size-independent measure of whether the brain tissue was compressed or stretched in the plane of analysis. A value of one for normalised brain tissue area means that brain tissue area did not change with time.

### Statistical analysis

Mean and standard deviation of brain structure size and tissue stiffness measurements were calculated for both the control and hydrocephalic rats. Firstly, the effect of time, group and time*group on (1) brain structure size (i.e cross-sectional area of the ventricles, cranium, and caudate-putamen, and the thickness of the cortical gray matter); and (2) brain stiffness (G* cortical gray matter and caudate-putamen) were examined for both the control and adult hydrocephalic rats using generalized estimating equations (GEE) that accounted for the repeated measures design. This was followed by pairwise comparisons to compare the control and hydrocephalic rats at each time-point and the two time-points between each group of rats. Secondly, for adult hydrocephalic rats, the effect of ventricle size on brain structure sizes and stiffness were tested using GEE, also accounting for repeated measures. Finally, comparison between the results in juvenile hydrocephalic rats and the adult hydrocephalic rats was made. The effect of group (adult vs juvenile hydrocephalic rats) on brain structure sizes, stiffness and the normalised brain tissue area was assessed using a Mann-Whitney test. The linear relationships between normalised brain tissue area and changes in brain structure size and stiffness with respect to their baseline conditions were assessed using Spearman correlations for the hydrocephalic adult rats, juvenile rats and all rats (adults and juveniles). All statistical analyses were performed using SPSS (v22, IBM, Armonk, New York, USA), except for the Spearman correlations and t-tests which were calculated in GraphPad Prism (v6.01, GraphPad Software Inc, La Jolla, CA, USA). For all tests, P < 0.05 was considered significant.

## Results

All animals survived and tolerated the sham injection or surgery except for one hydrocephalic rat which developed subarachnoid haemorrhage after the surgery. The animal was euthanized one day later (two days before the end-point). Data from this animal obtained prior to surgery is included in the results and statistical analysis. The hydrocephalic rats lost an average of 12 ± 5% of their body weight after the surgery: (day of surgery: 241 g ± 19 g, day three: 211 g ± 27 g). In contrast, the weight of the control rats did not change (day of surgery: 229 g ± 15 g, day three: 230 g ± 14 g).

### The effect of time in adult hydrocephalic and control rats

**Fig 2:**
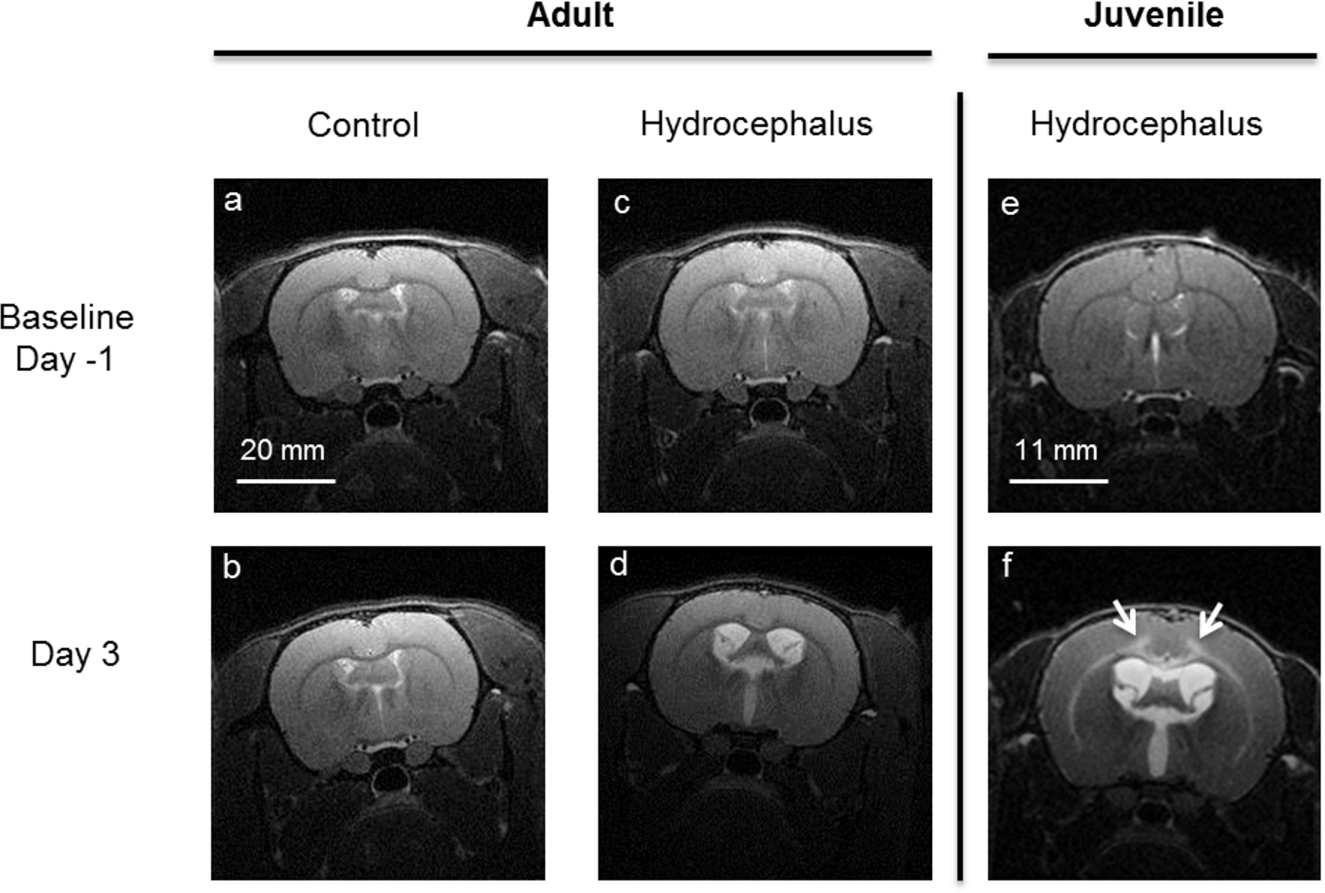
Typical axial high resolution T2-weighted anatomical MR images of adult and juvenile rats. Control (a,b) and hydrocephalic adult rat (c,d) at baseline (Top row, one day prior to injection) and three days post-injection (bottom row). Kaolin injected into the cisterna magna enlarged the ventricles in the hydrocephalic rats. The size of the ventricles did not change in the sham injected controls. Fig 2 (e,f) show a typical MRI image of a juvenile rat at baseline and 3 days post injection. In the juvenile rats, the hyper-intense signal (see white arrows in f) was attributed to the presence of oedema in the white matter and inner layers of the cortical gray matter. In contrast, this was not observed in the adult hydrocephalic rats (see d). Brightness of all images was increased by 20% for visualization.

**Fig 3:**
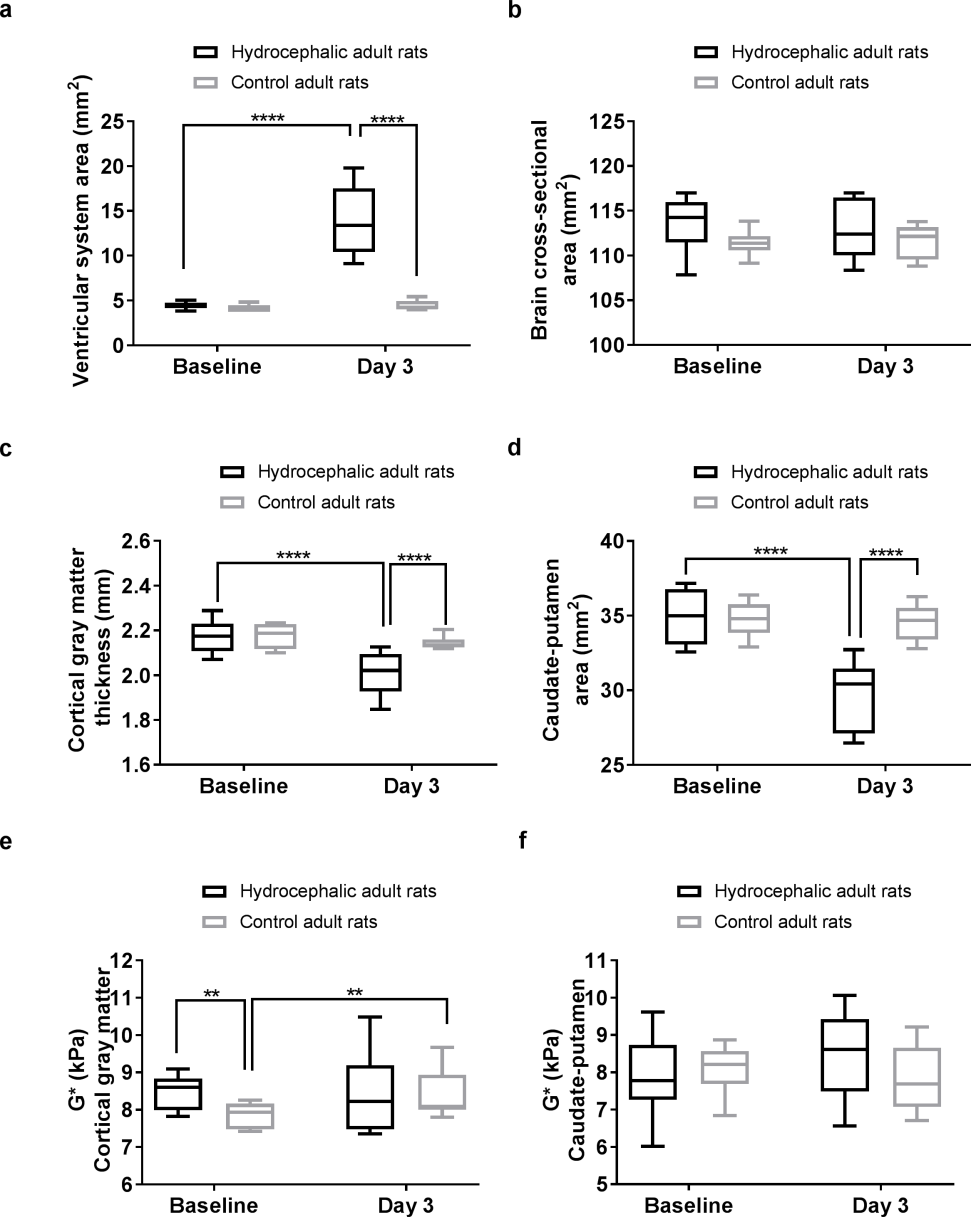
Brain tissue deformation and stiffness during hydrocephalus development in adult rats. Three days post hydrocephalus induction, the hydrocephalic rats had significantly (a) larger ventricular system cross-sectional area, (b) larger cranial cross-sectional area, (c) smaller cortical gray matter thickness, and (d) smaller caudate-putamen cross-sectional area, but there were no significant changes in (e) cortical gray matter stiffness and (f) caudate-putamen stiffness. Significant pairwise comparisons are reported on each graph.

Fig 2A, 2B, 2C and 2D shows a typical set of anatomical images for the hydrocephalic and control rats at baseline and three days post-injection. The mean and standard deviation of the brain tissue deformation and stiffness at each time point are reported in Fig 3 and in S1 Table (S1 Table) for both groups.

In the hydrocephalic rats, the cortical gray matter thickness and the caudate putamen cross-sectional area decreased over time. There were no changes in the controls. The cranial cross-sectional area did not change with time in either group.

Stiffness of the cortical gray matter and the caudate-putamen in the hydrocephalic rats and the stiffness of the caudate-putamen in the control rats did not change over time. No significant differences were observed in the caudate-putamen stiffness between the two groups at either time point nor in the cortical gray matter stiffness three days post injection. However, the cortical gray matter stiffness in controls was lower than the hydrocephalic rats at baseline (P = 0.004) but was similar to the hydrocephalic rats (~8.5 kPa) on day 3. There was no significant time by group interactions for the cortical gray matter or caudate-putamen stiffness in both the adult hydrocephalic and control groups, but an increase in stiffness with time was observed in the cortical gray matter in controls.

### The effect of ventricle size in adult hydrocephalic rats

**Fig 4:**
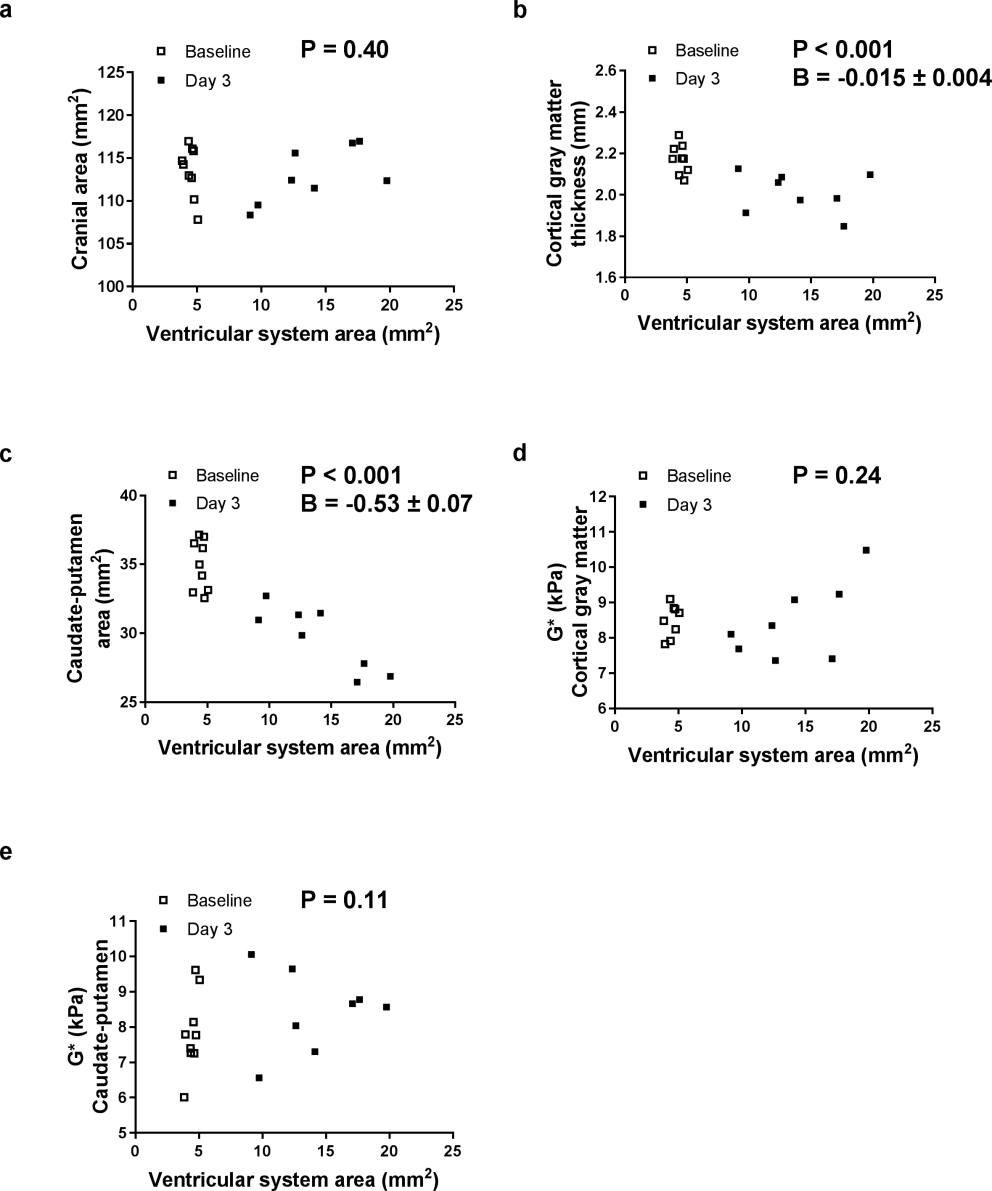
Effect of ventricular enlargement on brain tissue deformation and stiffness in adult hydrocephalic rats. Relationships between the ventricle system cross-sectional area and (a) the cranial cross-sectional area, (b) cortical gray matter thickness, (c) caudate-putamen cross-sectional area, and shear modulus (G*) of the (d) cortical gray matter and (e) caudate-putamen in adult hydrocephalic rats. Relationships were evaluated using generalized estimating equations accounting for the repeated measures. For relationships that were significant, the parameter B represents the increase in that parameter per mm^2^ of ventricle system area.

When the ventricles enlarged, the cortical gray matter thickness and the caudate-putamen cross-sectional area decreased (P < 0.001 for both) but the cranial cross-sectional area and both the cortical gray matter and caudate-putamen stiffness did not change (Fig 4).

### Brain tissue deformation and stiffness comparison between adult and juvenile hydrocephalic rats

Three days post kaolin injection, the area of the ventricles was 3.1 ± 0.9 times larger in the adult hydrocephalic rats and this was significantly lower (P = 0.01) than the ventricular enlargement in the juvenile hydrocephalic rats (4.1 ± 0.4 times compared to baseline). In addition, hyper-intense signal in the cortical gray matter on the T2 weighted images, which indicated the presence of oedema in juvenile hydrocephalic rats, was not observed in adult hydrocephalic rats (Fig 2E and 2F) [1].

**Fig 5:**
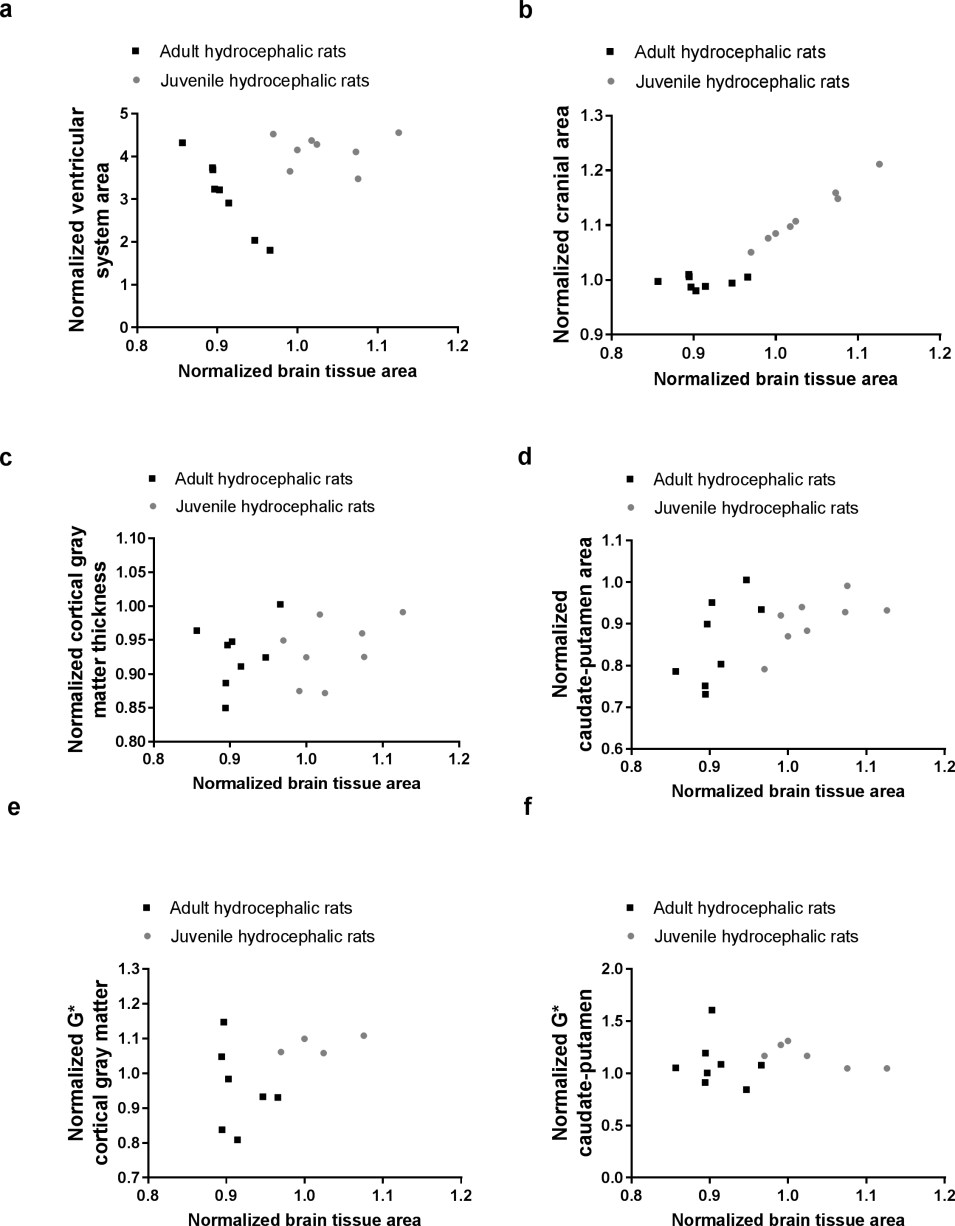
Effect of the brain compression on brain tissue deformation and stiffness in adult and juvenile hydrocephalic rats, three days post-hydrocephalus induction. Relationships between the normalised brain tissue area and (a) normalised ventricular system cross-sectional area, (b) normalised cranial cross-sectional area, (c) normalised cortical gray matter thickness, (d) normalised caudate-putamen cross-sectional area, and normalised shear modulus (G*) of the (e) cortical gray matter and (f) caudate-putamen. Relationships were evaluated with Spearman correlations (Table 1). Note: In juvenile rats, four normalised G* measurements of the cortical gray matter and two of the caudate-putamen could not be calculated because the measurement was missing either at baseline or day 3.

In the presence of enlarged ventricles, between the adult and juvenile hydrocephalic rats, neither cortical gray matter thickness (P = 0.49, 2.0 ± 0.1 mm, 2.1 ± 0.1mm, respectively), nor the caudate-putamen cross-sectional area (P = 0.16, 30 ± 2 mm^2^, 31 ± 2 mm^2^, respectively) were significantly different. However, normalised brain tissue area in the adult hydrocephalic rats (0.91 ± 0.03) was significantly lower than in the juvenile hydrocephalic rats (1.03 ± 0.05, P = 0.0002). This suggests that the brains of the adult hydrocephalic rats were more compressed although their ventricles enlarged less than the juvenile hydrocephalic rats. Results also showed that across all rats (juveniles and adults) when the normalized brain tissue area decreased, the normalized cranial and caudate-putamen cross-sectional area decreased (Fig 5B and 5D) (Table 1).

**Table 1:** Statistical results from Spearman correlation analyses between normalised brain tissue area and changes in brain structure size and stiffness with respect to their baseline conditions. Data is presented graphically in Fig 5.

*Spearman correlations* *(P—values)*	All rats	Adults only	Juveniles only
Normalised ventricular system area	P = 0.20	P< 0.0001,r = -1	P = 0.98
Normalised cranial area	P < 0.0001,r = 0.84	P = 0.46	P = 0.0004,r = 0.98
Normalised cortical gray matter thickness	P = 0.35	P = 0.46	P = 0.33
Normalised caudate-putamen area	P = 0.045,r = 0.25	P = 0.046,r = 0.74	P = 0.06
Normalised G* cortical gray matter	P = 0.25	P = 0.40	P = 0.75
Normalised G* caudate-putamen	P = 0.65	P = 0.93	P = 0.10

At baseline, both the caudate putamen and the cortical gray matter stiffness were stiffer in adult than in juvenile hydrocephalic rats (Caudate putamen, adults 7.8 ± 1.1 kPa, juveniles 6.0 ± 0.3 kPa, P = 0.002; Cortical gray matter, adults 8.5 ± 0.5 kPa, juveniles 7.0 ± 0.2 kPa, P = 0.0003). Three days post hydrocephalus induction, the caudate-putamen was stiffer in adults than in juveniles (adults 8.5 ± 1.2 kPa, juveniles 6.9 ± 0.5 kPa, P = 0.01), while the cortical gray matter was not significantly different between groups (adults 8.5 ± 1.1 kPa, juveniles 7.7 ± 0.2 kPa, P = 0.22). Finally, when the brain tissue stiffness was normalized to baseline values, no significant relationship was observed with the normalized brain tissue area (Fig 5E and 5F).

## Discussion

Acute hydrocephalus in adult rats was successfully induced in this study, including compression of the brain tissue, as demonstrated by reduced cortical thickness and deep gray matter cross-sectional areas. However, brain tissue stiffness in the adult hydrocephalic rats was not significantly higher than controls and stiffness was not associated with the size of the enlarged ventricles. When the data from the current study were analysed together with the results of juvenile hydrocephalic rats obtained from a previous study, the relative change in brain stiffness from baseline was not correlated with normalised brain tissue area.

There was a rapid increase in brain ventricle size in the adult hydrocephalic rats after the induction of hydrocephalus and this was consistent with our previous study on juvenile hydrocephalic rats [1]. However, the rate of ventricular enlargement was faster in the juvenile rats and this was likely because the circumference of the brain tissue was able to stretch with the cranium due to the open cranial sutures. In the adult hydrocephalic rats, skull volume expansion was not possible due to the closed skull sutures and the dilated brain ventricles expanded the deeper brain tissues outwards, which narrowed and compressed the subarachnoid space, and the cortical and the caudate putamen regions against the surrounding skull. Similar to the juvenile hydrocephalic rats in [1], brain ventricle enlargement was associated with reduction in cortical gray matter thickness and decrease in caudate putamen cross-sectional area. However, these anatomical changes were not associated with changes in brain tissue stiffness in the adult hydrocephalic rats and this was in stark contrast to the juvenile hydrocephalic rats, which had increased in caudate putamen stiffness at day three, scanned using the same MR protocol. In the previous study on juvenile hydrocephalic rats, there was a non-significant trend towards increased stiffness in the cortical gray matter and this was likely related to the emergence of oedema in the cortex, which may have counterbalanced the mechanical stiffening effect of tissue compression [14]. Similarly, the absence of significant change in brain tissue stiffness in the adult hydrocephalic rats in this study may also be related to a few factors that have counterbalanced the increase in brain stiffness. For example, brain stiffness in the adult hydrocephalic rats could have been offset by a milder oedema not pronounced enough to be visible on the T2 weighted anatomical scans. Other possible confounding factors include the emergence of tissue necrosis [1,15], demyelination [12] and alterations to the local blood vessel architecture [16] due to potentially higher intracranial pressure in the adult hydrocephalic rats compared to the juvenile hydrocephalic rats. Further investigations of changes in brain tissue microstructure will likely be useful [17,18] in providing further insight into why in-vivo brain stiffness did not change significantly in the adult hydrocephalic rats with closed skull sutures.

There were two unexpected observations in this study. Firstly, the cortical gray matter stiffness between the control and adult hydrocephalic rats at baseline was significantly different. Although high incidence of spontaneous ventriculomegaly in rats has been reported [19], there were no abnormalities visible on the anatomical images of the control and adult hydrocephalic rats in this study. Reasons for the difference in baseline cortical gray matter stiffness between the hydrocephalic and control rats is unclear but is likely related to a type I error (i.e. spurious false positive). There were no deviations in the rats’ age, where they were purchased, or their living conditions prior to the scan. Indeed, the rats were randomly allocated to the control and hydrocephalic groups. In addition, rats in the control and hydrocephalic groups were also scanned on the same day and were therefore unlikely to be affected by any slight differences in the experimental setup. Secondly, the adult control rats showed an unexpected increase in cortical gray matter stiffness with time and this is unusual since brain tissue stiffness and microstructure were not expected to change substantially in animals of this age. This discrepancy was likely related to the abnormally low baseline cortical stiffness values in the controls and further studies with larger sample size may help to verify this.

Results from this study show that changes in brain tissue mechanical properties and their underlying mechanisms in hydrocephalus are complex and likely dependent on their phenotypes. While existing MRE studies of humans with NPH [9,10] demonstrated significant differences in brain tissue stiffness, results from this study show that compressed brain tissue in hydrocephalus is not necessarily associated with increases in brain stiffness. Contrary to expectation, the brain tissue stiffness did not vary with the size of the enlarged ventricles in either the adult or juvenile hydrocephalic rats. Therefore, although MRE is an attractive imaging modality that may be used to improve the diagnosis and monitoring of hydrocephalus, further knowledge of how brain tissue stiffness and microstructure change immediately after the onset of the disease and over time will be required before this MR technique can be applied routinely in the clinic for diagnosis or monitoring of treatment outcomes. In addition, as changes in stiffness and tissue microstructure could be subtle between disease states and severities, future studies will require larger sample sizes to quantify any smaller effect size to ascertain the effectiveness of MRE as a diagnostic tool for hydrocephalus.

### Limitations

There were several inherent limitations in this study that need to be considered. Firstly, the spatial resolution of the MRE data was slightly lower in this study (350×350×350μm^3^) than what was used for the previous study on juvenile hydrocephalic rats (300×300×300μm^3^). This small difference was unlikely to have affected the stiffness measurements and comparisons between the two groups of animals. Secondly, the MRE reconstruction was performed under the assumption that the brain is isotropic. While anisotropic reconstruction for MRE has been successfully performed in muscles [7,15], it is still under development for brain tissue. Thirdly, as previously mentioned, multiple pathological events that influence tissue stiffness could have taken place concurrently and additional imaging techniques would have been useful to clarify the mechanisms. For example, in the previous study on juvenile rats, demyelination was assessed by the change in radial diffusivity [1] in diffusion tensor imaging (DTI). Additional imaging techniques such as DTI were not performed in this study because the study design was limited by the well-being of the adult hydrocephalic rats which deteriorated more quickly than the juvenile hydrocephalic rats (potentially due to higher intracranial pressure) in our preliminary studies. Similar reasons have limited the study of hydrocephalus development in adult rats over a longer time period to match the previous work on juvenile hydrocephalic rats. Other rodent hydrocephalus models, such as those described in [20], may potentially allow animals to survive longer with the disease and enable studies of how brain tissue stiffness changes at later time points. Fourthly, intracranial pressure (ICP) in the adult and juvenile hydrocephalic rats was not measured because ICP measurement is invasive and was not compatible with the MRE mechanical setup. ICP measurements may shed light on whether this could influence brain stiffness measurements in the disease. Finally, brain deformation was represented by normalised brain tissue area in the plane of analysis to limit scan time, although change in brain tissue volume would be a better indicator for brain tissue deformation as this would allow for any out of plane deformation of the brain to be considered.

## Conclusions

This study shows that the cortical and caudate-putamen stiffness in an adult rat model of obstructive hydrocephalus did not change significantly during ventricular enlargement although brain tissue in the rats was significantly compressed. These results were in contrast with our previous findings in juvenile hydrocephalic rats, whose brains stiffened despite a less compression. The latter may have occurred because the brain circumference was able to stretch with the cranium due to the open skull sutures. This suggests that while MRE has the potential to be used as a tool for tracking brain biomechanics in hydrocephalus, and may be useful for differentiating hydrocephalus from other brain diseases which have ventriculomegaly but without any brain compression, further studies of the interactions between brain stiffness, brain tissue deformation, tissue oedema and neural damage are necessary to enhance our understanding of its use in hydrocephalus.

## Supporting information

S1 Table Mean and standard deviation for brain deformation and stiffness measures in hydrocephalic (HCP) and control rats at baseline (day-1) and three days post injection (day 3). (DOCX)
