# Supplementary material for: Development of acute hydrocephalus does not change brain tissue mechanical properties in adult rats, but in juvenile rats
Source: PLoS One. 2017 Aug 24;12(8):e0182808. doi: 10.1371/journal.pone.0182808 (PMC5570303; doi:10.1371/journal.pone.0182808)
Supplement: S1 Table — (DOCX) [file pone.0182808.s001.docx]

| Brain structure | Variables | Group | Baseline | Day 3 | P _time *group_ | Multiple comparisons (P values) | | |
| --- | --- | --- | --- | --- | --- | --- | --- | --- |
|  |  |  |  |  |  | Baseline vs day 3 | HCP vs Control at Baseline | HCP vs Control at day 3 |
| Ventricles | Ventricular system cross-sectional area of the (mm^2^) | Control | 4.2±0.4 | 4.4±0.6 | <0.001 (****) | 0.41 | 0.13 | <0.001 (****) |
|  |  | HCP adult | 4.5±0.4 | 14.1±3.8 |  | <0.001 (****) |  |  |
| Head | Cranial cross-sectional area (mm^2^) | Control | 111±2 | 112±2 | 0.35 | 0.73 | 0.05 | 0.24 |
|  |  | HCP adult | 114±3 | 113±3 |  | 0.24 |  |  |
| Cortical gray matter | Thickness (mm) | Control | 2.18±0.05 | 2.14±0.03 | 0.001 (***) | 0.06 | 0.91 | <0.001 (****) |
|  |  | HCP adult | 2.17±0.07 | 2.01±0.10 |  | <0.001 (****) |  |  |
|  | G* (kPa) | Control | 7.8±0.4^#^ | 8.4±0.7 | 0.25 | 0.004 (**) | 0.003 (**) | 0.88 |
|  |  | HCP adult | 8.5±0.5^#^ | 8.5±1.1 |  | 0.99 |  |  |
| Caudate-putamen | cross-sectional area (mm^2^) | Control | 35±1 | 35±1 | <0.001 (****) | 0.76 | 0.78 | <0.001 (****) |
|  |  | HCP adult | 35±2 | 29±2 |  | <0.001 (****) |  |  |
|  | G* (kPa) | Control | 8.1±0.7 | 7.8±0.9 | 0.13 | 0.42 | 0.56 | 0.23 |
|  |  | HCP adult | 7.8±1.1 | 8.5±1.2 |  | 0.20 |  |  |

^#^ For one hydrocephalic and one control rat, G* cortical gray matter measured at baseline was excluded due to abnormal wave amplitude (7.2 μm and 2.4 μm, respectively). “*” represents P <0.05, “**” represents P < 0.01, “***” represents P < 0.001, and “****” represents P < 0.0001.
